# Notes and News

**Published:** 1893-12-23

**Authors:** 


### Notes and News.

Collected and Communicated.

It is to America that women physicians owe their status in Turkey. Until the United States Minister, Judge Terrell, successfully petitioned on their behalf, women could not hold a recognized diploma in Turkey. An increasing number of female physicians have carried on their calling without legal support, a large number being American medical missionaries. It is therefore a suitable circumstance that they will owe their improved condition to their own countryman.

Whilst the limited spaces on our small island are yearly further encroached upon by a growing population, the vast lands of Russia show signs of a diminishing habitation. A census is talked of next year, none having been taken since the year 1857, and then an opportunity will be afforded of judging as to the accuracy of the deductions from the present health reports. These health statistics are taken annually, and the last published one, that for the year 1890, shows a marked falling off in the natural increase of population and an unusually high death-rate. Russia has been heavily scourged with disease during the last few years, and this and the condition of poverty account mainly for the dwindling population.

The exhibition of the "Gothic," White Star Line steamship, on Saturday in aid of the Seamen's Hospital was a great success. Crowds proceeded to the Royal Albert Docks throughout the day, and explored the ship with a great amount of interest. The vessel displayed all her bunting, and the attendant band added to the brightness of the scene. Doubtless few of the visitors on Saturday had seen before one of our ocean palaces. In fittings and arrangement the "Gothic" has had the advantage of every modern improvement, and the sleeping accommodation is such as to rob ocean travelling of all idea of discomfort. Brass bedsteads, pretty curtains, and polished mahogany furniture are supplied to the most privileged, whilst the rest of the state rooms are but a little less finished in arrangement. The gold-and-white of the saloons adds to the general bright and cheery effect of the whole ship. The bath rooms struck the sanitary critic as admirable, and we wished every hospital the possessor of such excellent porcelain baths. About £500 was reeeived.

The annual meeting of the constituents of the Hospital Sunday Fund was held on Tuesday, the Lord Mayor in the chair, whose reappearance after his illness was greeted with cheers. The usual formal business was transacted, and Sunday, June 10th, was, on the motion of Archdeacon Sinclair, fixed as the date for Hospital Sunday, 1894.

In his reply to the deputation versus the erection of an infectious diseases hospital at Kingston, the Duke of Cambridge has set an example of practical common sense which it is to be hoped will have a widespread influence for good. The Duke refused to give his name to the petition against the project on public and private grounds. He accepts the verdict of the military medical authorities that the health of the soldiers in barracks is not likely to suffer, and personally he does not fear the deterioration of his own property.

Mr. Justice North made a decision now confirmed by Lords Justices Lindley and Davey of importance to hospitals. The hospital in question is the Brompton Hospital for Consumption. A will in favour of the institution was made before the passing of the Mortmain Act, but came into operation subsequently. Mr. Justice North's verdict in favour of the hospital was appealed against by other legatees, and the confirmation of his decision by the Court of Appeal decides a point which would probably have been frequently raised without it.

It is impossible to ignore the fact that influenza has once more established its sway amongst us. The popular criticism on this visitation is, it is not of so virulent a type as that of the former epidemics. We are inclined to think facts show that to those above middle age it is equally fatal, the younger of the population, as on previous occasions, making a better fight against it. In Liverpool it is noticeable that the unusually large number of deaths from affections of the lungs is attributable to influenza, and that the majority of victims were the aged. As the health returns come in from various parts of the country, it is probable that the conditions will be found very much the same everywhere.

The small-pox and scarlet fever epidemics at Birmingham have caused a very strong light to be thrown on the arrangements made by the Health Committee. Through the instrumentality of this Board a permanent fever hospital, with accommodation for ninety-five patients, is being erected at Yardley, the cost of which is estimated at £38,000. In the meanwhile the present epidemics have broken out and demand provision for treatment. The Lodge Road Hospital has therefore been supplemented by temporary wards and the utilisation of an empty house, at an expenditure of upwards of £800. Large as this sum appears, many of the inhabitants of Birmingham contend that such a cost, occurring in time of epidemic, is far less than that to be imposed upon them as a definite annual payment by the establishment of a permanent fever hospital. Semi-permanent hospitals, the erection of which costs far less than that of permanent buildings, have proved quite satisfactory elsewhere. Moreover, the Chairman of the Health Committee appears to contemplate the erection of temporary wards in times of severe epidemics, in addition to the two permanent hospitals which Birmingham will then possess. No provison has been made that the new hospital is to be retained for either small-pox or scarlet fever patients exclusively, and it cannot be said that the inhabitants of the city are unreasonable in raising questions as to the advisability of so large an expenditure.

